# Gene Loss and Acquisition in Lineages of Pseudomonas aeruginosa Evolving in Cystic Fibrosis Patient Airways

**DOI:** 10.1128/mBio.02359-20

**Published:** 2020-10-27

**Authors:** Migle Gabrielaite, Helle K. Johansen, Søren Molin, Finn C. Nielsen, Rasmus L. Marvig

**Affiliations:** a Center for Genomic Medicine, Rigshospitalet, Copenhagen, Denmark; b Department of Clinical Microbiology, Rigshospitalet, Copenhagen, Denmark; c Department of Clinical Medicine, Faculty of Health and Medical Sciences, University of Copenhagen, Copenhagen, Denmark; d The Novo Nordisk Foundation Center for Biosustainability, Technical University of Denmark, Lyngby, Denmark; Emory University School of Medicine

**Keywords:** *Pseudomonas aeruginosa*, computational biology, evolution, genomics, host-pathogen interactions

## Abstract

Genome analyses have documented that there are differences in gene repertoire between evolutionary distant lineages of the same bacterial species; however, less is known about microevolutionary dynamics of gene loss and acquisition within bacterial lineages as they evolve over years. Here, we analyzed the genomes of 45 Pseudomonas aeruginosa lineages evolving in the lungs of cystic fibrosis (CF) patients to identify genes that are lost or acquired during the first years of infection. On average, lineage genome content changed by 88 genes (range, 0 to 473). Genes were more often lost than acquired, and prophage genes were more variable than bacterial genes. We identified convergent loss or acquisition of the same genes across lineages, suggesting selection for loss and acquisition of certain genes in the host environment. We found that a notable proportion of such genes are associated with virulence; a trait previously shown to be important for adaptation. Furthermore, we also compared the genomes across lineages to show that the within-lineage variable genes (i.e., genes that had been lost or acquired during the infection) often belonged to genomic content not shared across all lineages. In sum, our analysis adds to the knowledge on the pace and drivers of gene loss and acquisition in bacteria evolving over years in a human host environment and provides a basis to further understand how gene loss and acquisition play roles in lineage differentiation and host adaptation.

## Abstract (precis)

Bacterial airway infections, predominantly caused by P. aeruginosa, are a major cause of mortality and morbidity of CF patients. While short insertions and deletions as well as point mutations occurring during infection are well studied, there is a lack of understanding of how gene loss and acquisition play roles in bacterial adaptation to the human airways. Here, we investigated P. aeruginosa within-host evolution with regard to gene loss and acquisition. We show that during long-term infection P. aeruginosa genomes tend to lose genes, in particular, genes related to virulence. This adaptive strategy allows reduction of the genome size and evasion of the host’s immune response. This knowledge is crucial to understand the basic mutational steps that, on the timescale of years, diversify lineages and adds to the identification of bacterial genetic determinants that have implications for CF disease.

## INTRODUCTION

Gene acquisition and gene loss are prominent in bacterial evolution and are also crucial during adaptation to new environments (1, 2). In contrast to point mutations, small insertions and deletions (microindels), inversions, and translocations that gradually alter existing genomic content, the acquisition or loss of entire genes rapidly confer large changes to the genomic content which alter bacterial phenotypes such as virulence, antibiotic resistance, and metabolic capability (3, 4). Thus, genome-wide analysis of the gene presence or absence is necessary to better understand bacterial evolution and adaptation (5).

While genome comparison of evolutionarily distant lineages of the same bacterial species gives insight into gene flux over the macroevolutionary scale, there is less knowledge of the pace at which and mechanisms by which genes are lost and acquired at the scale of microevolution, i.e., from studies of evolution of individual bacterial lineages (6, 7). Additionally, we have only a limited understanding of how lineage gene loss and acquisition are driven by selective versus genetic drift (1, 2).

Evolutionary studies on individual bacterial lineages are dependent on the ability to obtain multiple samples of the same lineage, which can be difficult in natural, *in vivo* environments that constantly change (8, 9), so studies are more easily performed *in vitro* (10–14). However, Pseudomonas aeruginosa infections in cystic fibrosis (CF) patients represent an infectious disease scenario in which the genomic evolution of individual bacterial lineages can be followed over the years and thus give an opportunity to research bacterial evolution and adaptation *in vivo* in the human host (15, 16). There is already a large pool of knowledge on the role of point mutations and microindels in evolution and adaptation of P. aeruginosa in CF patients, whereas gene loss and acquisition have been less extensively investigated (17–19). A better understanding of the genetic changes responsible for P. aeruginosa pathogenicity in CF patients is crucial to improve CF treatment strategies (20–22).

To better understand the role of gene loss and acquisition in within-host evolution and adaptation, we used genomic data from 474 longitudinally collected isolates of P. aeruginosa from children and young CF patients to investigate gene loss and acquisition in lineages of P. aeruginosa as they evolve from the initial invasion of CF airways and onward as they adapt to the human host. In total, 34 patients and 45 different clonal lineages were analyzed, and we aimed to identify gene loss or acquisition events in each of the different lineages to detect patterns across lineages ultimately leading to a better understanding of the genetic basis of bacterial adaptation in the human host.

## RESULTS

### *De novo* genome assembly and gene annotation.

We previously generated short-read sequencing data for the genomes of 474 isolates of P. aeruginosa sampled from the airways of 34 young CF patients to follow the genomic evolution of bacterial lineages within the host airways over the initial 0 to 9 years of infection (18). While the previous analysis aligned sequence reads to a P. aeruginosa reference genome to identify single nucleotide polymorphisms (SNPs) and small insertions and deletions (indels), we here used the same sequencing reads for *de novo* assembly of genomes to identify genes that are either lost or acquired during the course of infection.

**FIG 1:**
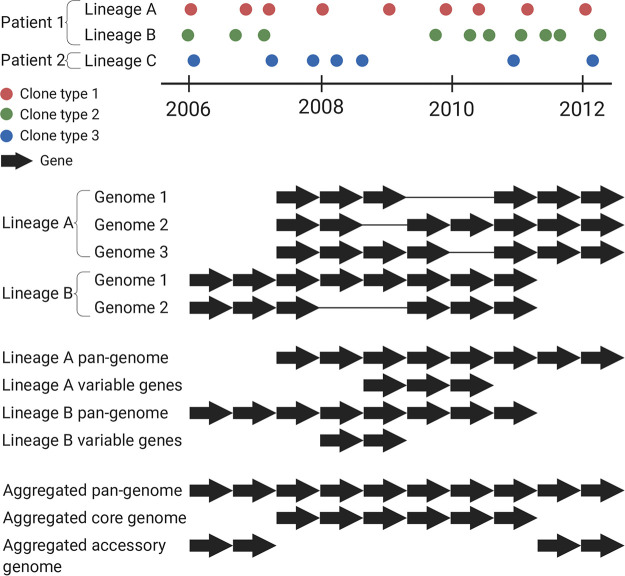
Schematic visualization of how bacterial lineages, lineage pan-genomes, within-lineage variable genes, and aggregated pan-genomes, core genomes, and accessory genomes were defined in this study.

We successfully *de novo* assembled the genomes of 446 isolates into 500 scaffolds or fewer (median, 172 scaffolds). The sizes of the assembled genomes ranged from 6,032,338 to 7,593,423 nucleotides (nt), and they contained 5,462 to 7,111 genes. The 446 assembled genomes represented 51 clone types as defined previously by Marvig et al. (2015) (18) (see Fig. S1 in the supplemental material). We grouped the isolates into 45 lineages; i.e., isolates of the same clone type and from the same patient were grouped together to allow identification of within-host accumulated gene differences (Fig. 1). In total, the 45 lineages encompassed 423 isolates distributed among 34 patients as 9 patients were infected with two (*n* = 7) or more (*n* = 2) clone types where multiple isolates were available (Fig. S1). The remaining 23 isolates with successful genome assembly were excluded from the analysis as there were no other clonal genomes available for the respective patients (*n* = 22) or the patient was infected multiple times with the same clone type and no other clonal genomes were available for that lineage (*n* = 1); i.e., at least two genomes were required for intralineage genome comparison.

10.1128/mBio.02359-20.2 FIG S1 Timeline and clone type of P. aeruginosa isolates from 34 CF patients included in the study. The clone types isolated only once are marked with circles. Download FIG S1, PDF file, 1.2 MB. Copyright © 2020 Gabrielaite et al. 2020 Gabrielaite et al. This content is distributed under the terms of the Creative Commons Attribution 4.0 International license.

### Pan-genomes and identification of gene presence-absence.

We analyzed 423 genomes in a two-step process to identify genes that showed variation within or between lineages. First, we compared the genomes of isolates of the same lineage to determine the full set of nonredundant genes found within the lineage, i.e., the lineage pan-genome. The lineage pan-genome consisted of (i) genes present in all isolates of the respective lineage (lineage core genome) and (ii) genes present in only some of the lineage isolates (lineage variable genes), i.e., genes that had been lost or acquired during the infection, referred to here as variable genes (Fig. 1). The lineage pan-genomes consisted of 5,607 to 7,008 genes longer than 150 bp, of which 0 to 473 were variable genes (median, 44 variable genes). A weak positive correlation (Pearson’s correlation coefficient 0.15, *P* value = 2.5 × 10^−3^) was identified between the assembly quality (number of scaffolds) and the number of absent genes (Fig. S2A) which did not explain the observed variability in gene content. Furthermore, by aligning the raw sequencing reads to the pan-genomes of the corresponding lineages, we determined that only 52 of 13,246 genes (0.4%) were incorrectly identified as absent by GenAPI because of a lack of assemblies. These genes were treated as present in all further analyses.

10.1128/mBio.02359-20.3 FIG S2 (A) Number of scaffolds in genome assemblies over the number of absent genes in 423 isolates used in gene presence-absence analysis. The blue line marks the linear regression estimate. (B) Heat map showing variable genes within lineage P98M3-DK36. Isolates are numbered according to date of sample. The earliest isolate is indicated as “Isolate 1.” (a) Multiple gene deletion during one deletion event. (b) Individual gene deletion. (C) Sequence alignment depth in early (blue) and late (peach) isolates in four lineages. The *x*-axis data correspond to the genomic position in the PAO1 reference genome. Red vertical lines mark the overlapping genetic regions of four lineages. (D) Histogram of pairwise SNP distances between P. aeruginosa isolates in the predefined core genome. The red vertical line marks the SNP distance threshold used by Pactyper. Download FIG S2, PDF file, 0.7 MB. Copyright © 2020 Gabrielaite et al. 2020 Gabrielaite et al. This content is distributed under the terms of the Creative Commons Attribution 4.0 International license.

**FIG 2:**
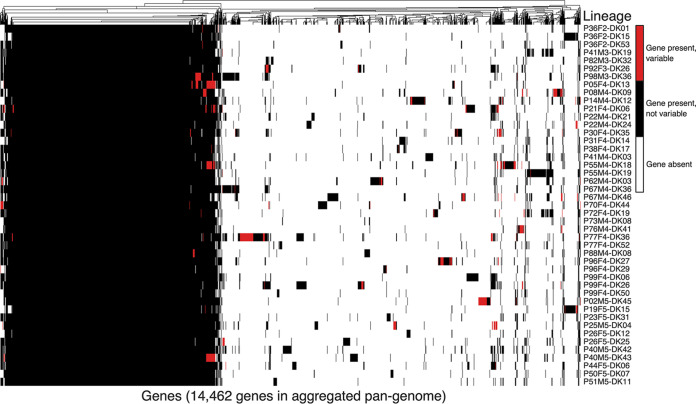
Presence or absence of 14,462 aggregated pan-genome genes in 45 lineages evolving in cystic fibrosis patients. Blue denotes that gene is present in all isolates of the lineage. Red denotes that the gene shows variable presence within the lineage. White denotes that the gene is not present in any of the isolates in the lineage.

Second, we compared the lineage pan-genomes to determine the full set of 14,462 nonredundant genes found across all lineages, i.e., the aggregated pan-genome (Fig. 1; see also Fig. 2). The aggregated pan-genome consisted of 4,887 genes shared across all lineage pan-genomes (aggregated core genome) and an aggregated accessory genome of 9,575 genes (genes present in only one or some lineage pan-genomes) (Fig. 1; see also Fig. 2). About half (4,932) of the aggregated accessory genes were unique for single lineages, and, overall, the lineage pan-genomes contained 0 to 540 (median, 78) of such lineage-specific genes (see Table S1 in the supplemental material). Furthermore, we found that all 335 genes reported to be essential genes in PAO1 and UCBPP-PA14 (23) were in the aggregated core genome; 29 of these genes were not present in one or more P. aeruginosa isolate genomes (Table S2).

10.1128/mBio.02359-20.5 TABLE S1 Table of pan-genome size and number of variable genes in aggregated core and accessory genomes among lineages. Also, findings of variable genes from Resfinder (antibiotic resistance) and from the VFDB database (virulence) and a pathoadaptive gene list (published previously by Marvig et al. [18]) are reported. Download Table S1, DOCX file, 0.02 MB. Copyright © 2020 Gabrielaite et al. 2020 Gabrielaite et al. This content is distributed under the terms of the Creative Commons Attribution 4.0 International license.

10.1128/mBio.02359-20.6 TABLE S2 A list of 29 essential genes (as defined by Liberati et al. [2006] [23]) and their PseudoCAP functions which were absent in one or several clinical isolates in this study. Download Table S2, DOCX file, 0.01 MB. Copyright © 2020 Gabrielaite et al. 2020 Gabrielaite et al. This content is distributed under the terms of the Creative Commons Attribution 4.0 International license.

Aggregated accessory genes were 15-fold more often variable within lineages than genes in the aggregated core genome (Table S1). While several factors might drive the higher turnover of aggregated accessory genes, one explanatory factor could be that the aggregated accessory genome has a larger amount of mobile genetic elements, such as prophage origin sequences. Therefore, we used the ACLAME database to identify and annotate phage and prophage sequences (longer than 150 bp) in the core and accessory genomes of the aggregated pan-genome, respectively. The accessory genome contained 116-fold more prophage genes, and these genes were highly variable over the course of infection; 58% of the prophage sequences in the accessory genome of the aggregated pan-genome were variable within lineages.

### Changes in gene content in lineages over the course of infection.

Next, we asked if the variable genes were either lost from or acquired in bacterial lineages. For this, we defined a gene as lost when it was present in the first isolate but absent in one or more of the later isolates and defined a gene as acquired when it was absent in the first isolate but present in one or more of the later isolates. Note that this definition of gene loss/acquisition might not be accurate as the first isolates might not represent the most recent common ancestor for the lineage. We found that the variable genes were more often lost. Of 3,955 variable genes, 3,411 were present in the first isolates and absent in the later ones, and the opposite was true for only 544 genes. Accordingly, we concluded that gene loss occurs at least 6 times more often than gene acquisition (Table S3).

10.1128/mBio.02359-20.7 TABLE S3 Counts of variable genes that were lost/acquired, variable individually or in a group, and of phage origin. Download Table S3, DOCX file, 0.02 MB. Copyright © 2020 Gabrielaite et al. 2020 Gabrielaite et al. This content is distributed under the terms of the Creative Commons Attribution 4.0 International license.

Prophage sequences and plasmids are known to be mobile elements in bacterial genomes. Prophage genes were found in all 45 lineages by using the ACLAME database. Prophage genes were among the variable genes in 22 of the lineages, and the prophage genes were lost in 70% of cases (Table S3); i.e., they were present in the early isolates and absent in the later ones. In contrast, plasmid genes were not identified to be lost or acquired in any lineage (the PlasmidFinder database was used to define plasmid genes). In total, three lineages (P41M3-DK19, P92F3-DK26, and P72F4-DK19) carried a plasmid belonging to the replicon IncQ2_1.

A total of 257 genes in the aggregated pan-genome were related to virulence as defined in the VFDB database. Of these, 17 genes were variable in at least one lineage, and in 8 of 17 cases, these genes were variable in more than one lineage. A two-sided Fisher’s exact test showed that virulence genes were in general less often lost/acquired than other genes (*P* value = 4.45 × 10^−9^). Furthermore, by using the Resfinder database, seven genes were defined to be related to antibiotic resistance in the aggregated pan-genome. None of these genes were lost/acquired in any lineages, while each isolate had 5 to 7 antibiotic resistance genes (Table S1). Of 52 pathoadaptive genes reported by Marvig et al. (2015) (18), 9 were lost/acquired in lineages. No significant difference in loss/acquisition between pathoadaptive and nonpathoadaptive genes was found by performing a two-sided Fisher’s exact test (*P* value = 0.861). Finally, we found that genes were 25-fold more often lost or acquired in a group than individually (see examples in Fig. S2B and Table S3); i.e., the loss/acquisition of 3,806 of 3,981 variable genes correlated with the loss/acquisition of other genes, while only 175 genes were lost/acquired alone.

### Convergent evolution: same genes are variable across lineages.

While variable genes made a small fraction of the aggregated pan-genome, and the majority of variable genes were lost/acquired in only one lineage, some genes were observed to be variable in multiple lineages.

**FIG 3:**
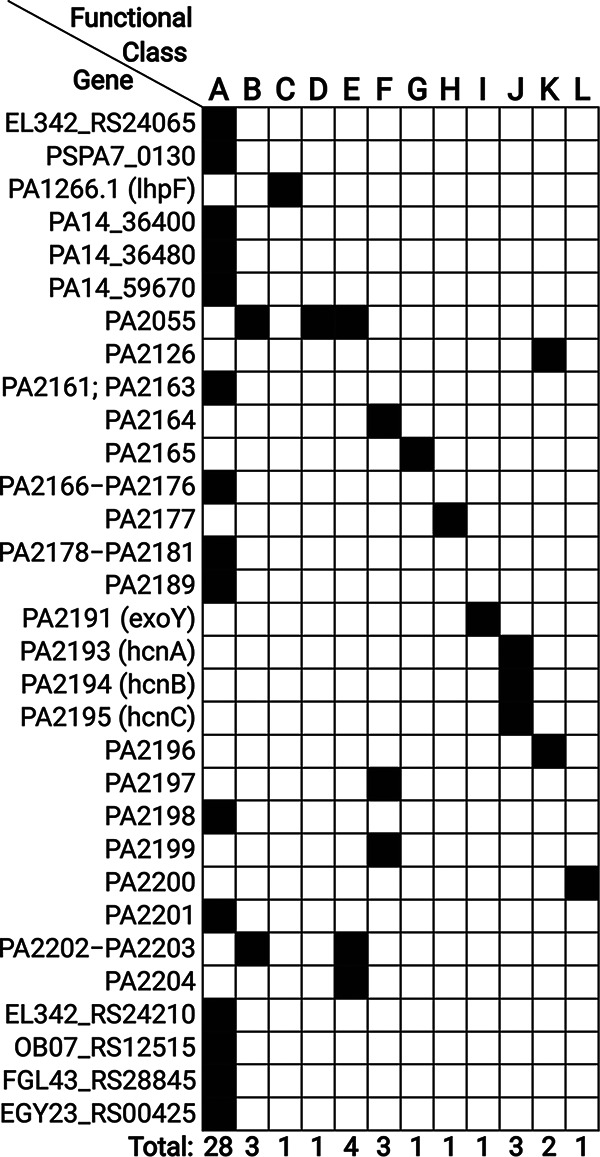
List of the most variable *Pseudomonas* origin genes and their function according to the PseudoCAP annotation. The genes code for proteins in the following categories: A—hypothetical, unclassified, unknown; B—membrane proteins; C—amino acid biosynthesis and metabolism related; D—antibiotic resistance and susceptibility; E—transport of small molecules; F—putative enzymes; G—energy metabolism; H—two-component regulatory systems; I—secreted factors (toxins, enzymes, alginate); J—central intermediary metabolism; K—transcriptional regulators; L—nucleotide biosynthesis and metabolism.

We defined genes as highly variable if they were identified as variable in ≥4 lineages (among the top 2% of all variable genes; Table S4). To ensure that the high level of variation was not due to technical artifacts of analysis, all highly variable genes were manually checked as follows: (i) P. aeruginosa origin genes were mapped to a PAO1 reference genome, and the coverage of the gene alignments was manually assessed; (ii) for other genes, the aggregated pan-genome was subjected to BLAST analysis by using the BLAST+ suite (24) against isolate genomes and then the alignments were manually assessed. Of the 54 genes initially identified as variable in ≥4 lineages, 2 were removed after the manual check (a detailed explanation is available in Materials and Methods). Of the 52 manually confirmed variable genes, 47 genes were variable in 4 lineages, 4 genes were variable in 5 lineages, and 1 gene was variable in 10 lineages (Fig. 3).

10.1128/mBio.02359-20.8 TABLE S4 Number of aggregated pan-genome genes that were variable in different numbers of lineages. Download Table S4, DOCX file, 0.01 MB. Copyright © 2020 Gabrielaite et al. 2020 Gabrielaite et al. This content is distributed under the terms of the Creative Commons Attribution 4.0 International license.

We annotated the highly variable genes according to PseudoCAP functional classes (25) (if present in PAO1/UCBBP-PA14 reference genomes) or by a BLAST search against the National Center for Biotechnology (NCBI) nucleotide collection (nr/nt) database. Most of the highly variable genes (34 of 52) were genes with hypothetical function or genes of non-*Pseudomonas* origin. The second-largest group of highly variable genes encoded membrane proteins (4 genes). Since genes encoding membrane proteins make up around 10% of the P. aeruginosa genome, we tested using a Fisher exact test if genes encoding membrane proteins are more variable than expected in accounting for their abundance, and we concluded that such was not the case (*P* value = 1.00). Other highly variable genes encoded proteins involved in amino acid and nucleotide biosynthesis, antibiotic resistance and susceptibility, transport, secreted factors, transcriptional regulation, and metabolism as defined in the PseudoCAP database (Fig. 3).

### Convergent evolution of locus with *hcnABC* and *exoY* genes.

**FIG 4:**
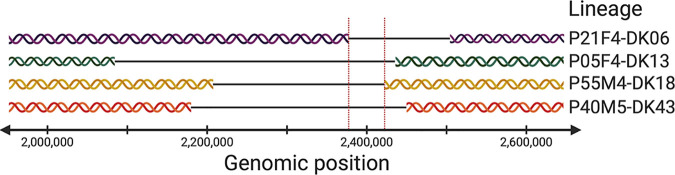
Genetic regions of four lineages where the same group of 34 genes is lost. Red vertical lines show the overlapping genetic regions lost in all four lineages.

We found that a group of 34 genes was lost/acquired in four lineages (P21F4-DK06, P05F4-DK13, P55M4-DK18, and P40M5-DK43). The 34 genes were orthologs of genes PA2161 to PA2181 and genes PA2189 to PA2204 in the PAO1 reference genome. We noted that three of the lineages (P05F4-DK13, P55M4-DK18, and P40M5-DK43) did not have genes PA2182 to PA2188 (genes flanked by PA2161 to PA2181 and genes PA2189 to PA2204 in the PAO1 reference genome) in their lineage pan-genomes and that genes PA2182 to PA2188 were variable and congregated with genes PA2161 to PA2181 and genes PA2189 to PA2204 in the fourth lineage (P21F4-DK06), so we concluded that the 34 genes were likely lost/acquired together rather than in separate two events (Fig. 4 shows the genetic region of the group of 34 variable genes). Further, we aligned reads from each of the lineages to the PAO1 reference genome to show that parallel deletion/insertion of the 34 genes was the result of larger yet different deletions/insertions in the individual lineages (i.e., the 34 genes represented the shared overlapping of four different deletions/insertions in the same genomic region; see Fig. S2C and Fig. 4).

In 3 of 4 lineages, genes were present in early isolates and absent in late isolates. For lineage P40M5-DK43, the 34 genes were present in the later of only two isolates that were sampled less than a year apart, so it is likely that two isolates represent different sublineages where one sublineage did not lose the genes while another one did. Also, genes PA2161 to PA2204 were present in all 45 lineage pan-genomes, suggesting that genes PA2161 to PA2204 were present in the ancestor of lineage P40M5-DK43 and thus were lost during the course of infection.

A total of 17 of the 34 genes were annotated as “Hypothetical, unknown or unclassified” by PseudoCAP; other genes were annotated as coding for “Putative enzymes” (4 genes), “Transport of small molecules,” “Membrane proteins,” “Central intermediary metabolism” (3 genes), or “Energy metabolism,” “Two-component regulatory systems,” “Secreted factors,” “Transcriptional regulators,” and “Nucleotide biosynthesis and metabolism” (1 gene) (Fig. 3). Some of these proteins had more than one function assigned by PseudoCAP. A literature search indicated that four of the genes (*hcnABC* and *exoY* encoding hydrogen cyanide synthase and type III secreted protein, respectively) are known to play a role in the virulence and pathogenesis of P. aeruginosa (25, 26).

### Convergent evolution in prophage-related genes and genomic islands.

A total of 6 of the 52 highly variable genes were identified as prophage origin genes originating from different P. aeruginosa prophages, similarly to the genes from phi1 and phi2 *Pseudomonas* phages. The variable groups of 56 to 82 genes which included the 6 most variable prophage genes might represent yet-undescribed genomic islands (GIs) as they are adjacent to tRNA encoding genes, contain both prophage origin and P. aeruginosa origin sequences, and are longer than 10,000 bp.

**FIG 5:**
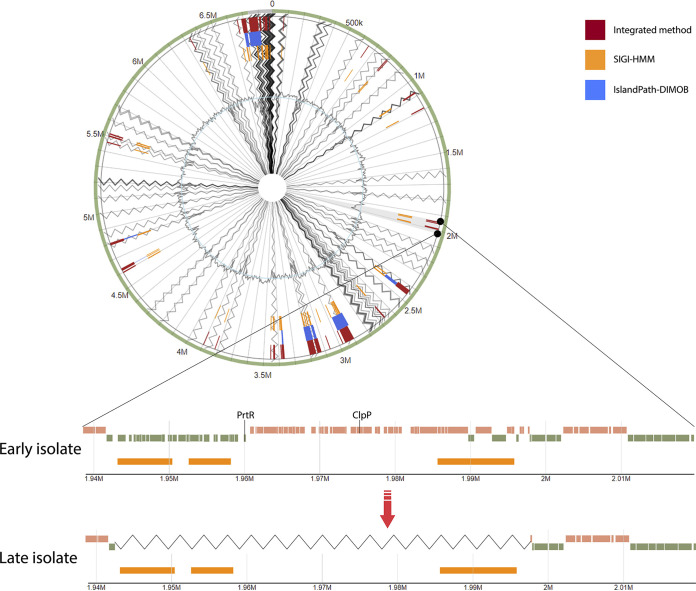
Genomic island predictions for early and late isolates of lineage P67M4-DK46. The zoomed region shows gene loss (zigzag line) in the late isolate. Possible virulence factors in the zoomed region are marked in the early isolate. Orange blocks in early and late isolates indicate predicted genomic islands.

As the mobility of genomic islands could explain the high variability of these gene regions, we predicted GIs with IslandViewer4. All six prophage origin genes were predicted to be part of GIs, e.g., exemplified by a 78-gene deletion in a genomic island encoding virulence factors in lineage P67M4-DK46 (Fig. 5). While IslandViewer4 predicted on average 40 GIs (range, 15 to 59) per lineage, we note that, as the analyzed genomes were not complete (i.e., in scaffolds), the GI prediction should be interpreted carefully; e.g., GIs were often predicted at the ends of scaffolds.

On average, 90% (range, 87% to 92%) of the genes in the predicted GIs code for hypothetical proteins. Therefore, it is difficult to define the function of most of the genes present in GIs. However, possible drivers of the loss of predicted GIs were identified; we identified homologs of genes coding for Clp protease (7 lineages) or of the *prtR* gene (5 lineages) as parts of predicted GIs which are known to be related to bacterial virulence and pathogenicity (27, 28). In addition, other probable virulence factors were identified in multiple lineages (Table S5).

10.1128/mBio.02359-20.9 TABLE S5 List of genes which were defined as related to virulence for lineages where genomic islands with frequently lost or acquired prophage genes were predicted. Download Table S5, DOCX file, 0.01 MB. Copyright © 2020 Gabrielaite et al. 2020 Gabrielaite et al. This content is distributed under the terms of the Creative Commons Attribution 4.0 International license.

### Overall population structure: SNP and gene distances.

**FIG 6:**
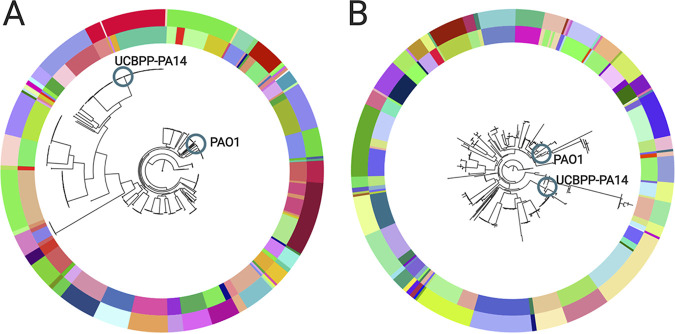
Phylogenetic trees of 446 P. aeruginosa samples (A) based on core genome SNPs and (B) based on gene presence-absence. The color of the outer circle of the trees denotes clone type, and the inner circle denotes the patient. Blue circles denote the position of reference genomes. The phylogenetic trees can be accessed on the Microreact webserver at https://microreact.org/project/KYbEXuFS0 (phylogenetic tree based on core genome SNP distances) and https://microreact.org/project/BkZdRqP-E (phylogenetic tree based on gene differences).

We wanted to understand our results determined for lineage genomes in the context of the overall population structure of P. aeruginosa. Accordingly, we determined the genetic relationships of the 446 isolates based on either SNPs in the core genome (Pactyper [see Text S1 in the supplemental material]) or gene presence-absence (GenAPI). Both the SNP-based and the gene-based phylogenies clustered the isolates according to clone type and patient (Fig. 6). Also, both phylogenies showed that the lineages clustered into one of two groups overall with either reference strain PAO1 or UCBPP-PA14, respectively. Furthermore, we confirmed that we obtained the same population structure (i.e., the same clustering according to clone type, patient, and reference strain) when we reconstructed the phylogeny with the native *de novo* assemblies as input and also when we masked recombined regions (4,570 of 224,614 SNPs [2%] were within the masked regions; Fig. S3).

10.1128/mBio.02359-20.1 TEXT S1 Description of Pactyper—a tool to define clone types and quantify genome SNP distances at the population level using whole-genome-sequencing (WGS) reads. Download Text S1, DOCX file, 0.02 MB. Copyright © 2020 Gabrielaite et al. 2020 Gabrielaite et al. This content is distributed under the terms of the Creative Commons Attribution 4.0 International license.

10.1128/mBio.02359-20.4 FIG S3 Phylogenetic trees of 446 P. aeruginosa samples without (A) and with (B) a recombination filter. The color of the outer circle of the trees denotes clone type, and the inner circle denotes the patient. Blue circles denote reference genomes. Download FIG S3, PDF file, 1.3 MB. Copyright © 2020 Gabrielaite et al. 2020 Gabrielaite et al. This content is distributed under the terms of the Creative Commons Attribution 4.0 International license.

**FIG 7:**
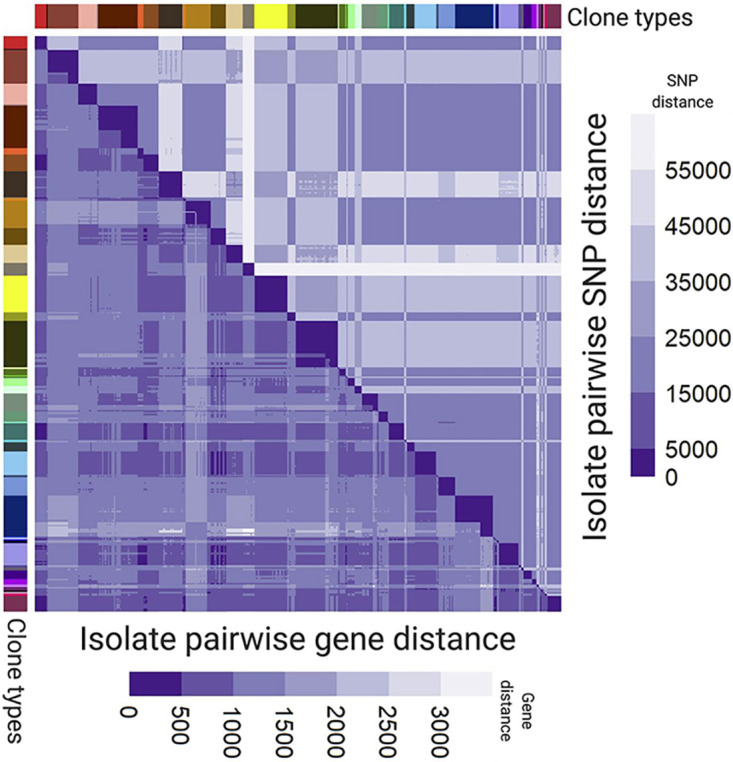
Pairwise SNP distances (top triangle) and gene distances (bottom triangle) between P. aeruginosa isolates with clone type annotation on the left side and on top.

The core genome pairwise SNP distance between lineages was on average 31,909 (22 to 67,325) SNPs, while the gene content difference was on average 1,142 (13 to 2,250) genes (one random isolate was chosen to represent each lineage to avoid overrepresentation of some lineages and underrepresentation of others) (Fig. 7). Moreover, the average diversities between lineages corresponded to 19,853 (22 to 24,957) SNPs and 1,043 (28 to 2,250) gene differences in the PAO1 group, while those within the UCBPP-PA14 group consisted of 35,191 (44 to 67,325) SNPs and 1,217 (13 to 1,775) gene differences. Performing Wilcoxon’s rank sum test on the distributions in the two groups, a significant difference between the groups was identified with a *P* value of <2.2·10^−16^ for pairwise SNP distance and a *P* value of 2.57 × 10^−13^ for pairwise gene difference distance, showing that the variability of SNPs and genes is lower within the PAO1 group than within the UCBPP-PA14 group.

Finally, we found that the ratios of core genome SNPs per difference in gene content were on average 3.5 (0.01 to 115.00; median, 0.66) and 30.2 (7.22 to 88.00; median, 28.95) within and between the clone types, respectively. Using a Wilcoxon’s rank sum test, we concluded that the difference between two groups is statistically significant, with a *P* value of <2.2 × 10^−16^.

### Reanalysis of other study data to compare sizes of pan-genomes and core genomes.

We analyzed publicly available genome sequencing data for a collection of 1,139 isolates that were previously included in a pan-genome and core genome analysis by Freschi et al. (2019) (6), to compare the pan-genome and core genome sizes between different collections of isolates. Using the same method as that used for our own isolate collection (GenAPI [29]), we found that the 1,139 isolates previously analyzed by Freschi et al. (2019) (6) shared a core genome of 2,360 genes within a pan-genome of 38,017 genes. Using the method of Freschi et al. (2019) (SaturnV), the core genome and pan-genome were shown to consist of 619 and 43,703 genes, respectively. Defining the core genome as consisting of all genes present in at least 99% of the samples, the core genome consisted of 4,870 and 3,879 genes for GenAPI and SaturnV, respectively.

## DISCUSSION

By analysis of genome sequences from 45 longitudinally sampled P. aeruginosa lineages from CF patients, we determined the microevolutionary dynamics of gene loss and acquisition in lineages of bacteria evolving in a human host environment. While similar analyses of within-host bacterial evolution investigated within-host gene loss and acquisition, our collection enables comparative analysis across multiple genotypically different strains (45 lineages distributed on 34 clone types) of the same species. Here, we not only identified events of gene loss or acquisition in the individual lineages but also analyzed this in the context of the gene variation across lineages, i.e., in the context of the species pan-genome and core genome.

### Pan-genomes and core genomes.

The aggregated pan-genome across lineages had 14,743 genes in total, and 4,887 genes were present in all 45 individual-lineage pan-genomes (i.e., those defined here as the aggregated core genome across lineages). Our findings are similar to reported from a study by Hilker et al. (2015) (30) that found the genomes of 21 P. aeruginosa strains to share a core of 4,748 genes of a pan-genome comprising 13,527 genes. In contrast, our calculated aggregated pan-genome and core genome sizes are significantly different from recent findings by Freschi et al. (2019) (6), where the pan-genomes and core genomes consisted of 54,272 and 665 genes, respectively. The difference may be explained in part by the fact that, while our isolates were collected only from CF patients in Denmark, the isolate collection used by Freschi et al. was more diverse. Also, by reanalysis of the data set from Freschi et al. with both our (GenAPI) and the original (SaturnV) methods, we show that the difference in the pan-genome and core genome sizes can in part be ascribed to differences in bioinformatics analyses as the pan-genome and core genome sizes reported by Freschi et al. converged toward findings from this and other studies (30–33) when we reanalyzed the data with GenAPI. We previously compared tools used for gene presence-absence identification (GenAPI, SaturnV, Roary, panX, EDGAR, Pandelos and BPGA) (29), and we suggest that the relatively small size of the core genome reported by Freschi et al. (665 genes) may in part be a consequence of false-negative calls of gene absence due to incomplete genome assemblies (34). Future studies based on long-read sequencing may overcome the issue of incomplete assemblies.

### Within-host gene loss and acquisition.

We found that genes were six times more often lost than acquired in lineages during within-host evolution. It remains unknown if the lack of gene acquisition is a consequence of limitations due to the availability of donor DNA, mechanisms of DNA uptake, or selection (either selection against the acquisition of genes or lack of selection for the acquisition of genes). Nonetheless, we found our results to be in line with previous hypotheses proposing that genomes are selectively reduced during the course of infection (34–36). Note that we defined a gene as lost if it was present in the first isolate but absent in one or more of the later isolates and vice versa. This definition of gene loss/acquisition might not be accurate as the first isolates may not represent the most recent common ancestor for the lineage, and as such, our analysis may be confounded by stochastic sampling of multiple coexisting sublineages. More sampling is required to resolve population heterogeneity (37–40).

Most of the genes that were lost or acquired within the host were part of the genome that was not shared across lineages (i.e., the aggregated accessory genome). The relative low turnover in the aggregated core genome of 4,887 genes shared by all lineages suggests that, while these genes are not essential *per se*, they may be generally important for survival under the conditions met by P. aeruginosa in the human host environment. Accordingly, they are maintained in the genomes. In contrast, 29 of the essential genes defined by Liberati et al. (2006) (23) were absent in some clinical isolates. This discrepancy could be explained by different conditions with respect to the human airways and laboratory media which were used in the study by Liberati et al. The lack of overlap of essential genes in different experiments reported previously by Poulsen et al. (2019) (41) corroborates the belief that *in vitro* experiments do not fully reflect the processes observed *in vivo*. Furthermore, some bacterial clones could compensate for a lost essential iron acquisition gene or antimicrobial resistance gene by cheating, i.e., by exploiting the molecules produced by other cells (42, 43).

In contrast, the prophage genes were the genes that were most often lost or acquired within the host, and prophages were putatively the major source of new genetic material. A total of 268 of 462 (58%) of the prophage genes in the aggregated pan-genome were variable within hosts, and despite taking up only 3% of the aggregated pan-genome, prophage genes constituted 9.4% of all within-host variable genes. This confirms the idea that prophage-facilitated gene flux is abundant and supports the conclusions from other studies indicating that prophages play an important role in P. aeruginosa CF infections (44, 45). The lack of plasmids and, therefore, their variability could be associated with the high fitness cost of carrying a plasmid in P. aeruginosa (46) and with the overall low number of identified plasmids in P. aeruginosa genomes (of 5,370 P. aeruginosa genomes in the NCBI database, only 70 have plasmids) (47). Finally, the absence of *Pseudomonas* plasmid annotations in the PlasmidFinder database could have led to a low number of identified plasmids among our isolates.

### Convergent evolution and adaptive loss of virulence.

The sampling from multiple lineages (and across multiple patients) allowed us to detect genes that were lost or acquired independently in parallel evolving lineages. While most genes were variable in only a single lineage, we found 52 genes to be variable within ≥4 lineages, which constitutes the top 2% most variable genes. The observed parallel loss or acquisition of the same genes across lineages may be driven by selection for loss and acquisition of certain genes in the host environment. It was previously hypothesized that virulence factors are selected against in CF infections, and in agreement with this, we found that 34 of the 52 highly variable genes were lost as part of a genomic region encoding the virulence factors hydrogen cyanide synthase (*hcnABC*) and type III secreted protein ExoY (*exoY*). It was also shown previously by Wee et al. (2018) (26) that selective pressures associated with loss of *hcnA*, *hcnB*, *hcnC*, and *exoY* genes exist, and Wee et al. also observed deletions of various sizes around the respective genes. Furthermore, the selective pressure associated with loss of *hcnABC* locus virulence genes was recently shown to possibly be related to the increased antibiotic resistance in multidrug-resistant strains (36).

We noticed that, while virulence genes *hcnABC* and *exoY* were among the most variable genes, in general, the virulence genes were less often variable within lineages. This may be counterintuitive if loss of virulence is beneficial for bacteria in chronic infections; nonetheless, we recognize that virulence factors may be downregulated rather than deleted as suggested previously by Rau et al. (2010) (44).

Genes were 25 times more often observed to be lost or acquired as groups of genes than as single-gene losses or acquisitions. This observation is in line with previous studies (35, 36) and illustrates how the presence of specific genetic elements enables and defines mobilization of genes: 6 of the 52 highly variable genes were prophage genes, and prophage regions often act as mobile elements. Accordingly, these six prophage genes were part of groups of 56 to 82 genes that were deleted together and constituted genomic islands.

All gene groups that were lost with the six highly variable prophage genes contained the gene orthologs coding for Clp protease as well as the *prtR* gene. PrtR is required for type III secretion system (28), and Clp protease induces virulence by regulating flagellar gene expression and ultimately increasing bacterial adhesion (27). Accordingly, the frequent loss of *prtR* and Clp protease genes adds to our observation that virulence factors are lost during infection. This loss of virulence may be positively selected in the host environments as the virulence factors activate the host immune response; hence, loss of virulence helps the bacteria to hide from the immune defense. Two of eight lineages with loss of Clp and *ptrR* genes also lost *hcnABC* loci and *exoY* genes, and as such, we observed no evidence that losses of the different virulence factors were mutually exclusive or concurrent (see Table S6 in the supplemental material).

10.1128/mBio.02359-20.10 TABLE S6 List of lineages where loss of virulence was observed through loss of Clp protease or loss of *Pseudomonas* origin genes (*hcnABC* loci and *exoY* gene). Download Table S6, DOCX file, 0.01 MB. Copyright © 2020 Gabrielaite et al. 2020 Gabrielaite et al. This content is distributed under the terms of the Creative Commons Attribution 4.0 International license.

### Population structure.

We described the population structure of our P. aeruginosa population of 446 isolates using both SNPs and gene absence/presence information, and in both ways, we identified two major phylogenetic clusters, one with PAO1 and one with UCBPP-PA14, in agreement with previous studies by Hilker et al. (2015) (30) and Stewart et al. (2014) (45). Furthermore, we showed that the levels of SNP and gene differences are significantly lower among PAO1-like isolates than among UCBPP-PA14-like isolates. Finally, we determined that there were significantly fewer SNPs per gene loss/acquisition in isolates belonging to the same clone type than in isolates from different clone types. We have previously shown for this data set that recombination of homologous DNA does not play a major role in microevolution within the CF host (18), and this in line with conclusions previously reported by Winstanley et al. (48). In contrast, it is likely that recombination of homologous DNA plays a relatively larger role over macroevolutionary scales in differentiating clone types and that such recombination plays a role in generating the larger amounts of differences in the number of SNPs per gene that we found in our comparisons of genomes across clone types.

Our study had several limitations. First, it is known that bacterial populations are highly heterogeneous across CF patient airway (49, 50); therefore, while we used the first isolate as a representative of the most recent common ancestor, that approach might not always have been valid. Furthermore, sequencing was mostly performed on single isolates from a sputum sample (218 of 312 cases), which additionally might have reduced the representation of true heterogeneity of bacterial lineages in the patient airway. To address these shortcomings, multiple isolates from each sputum sample should be sequenced. However, since we observed the same genetic variation tendencies across lineages, we believe that these limitations do not weaken the findings of this study. Short-read sequencing data were used in this study, which resulted in incomplete *de novo* assemblies increasing the uncertainty in gene loss and acquisition analysis. Nonetheless, we partly addressed this by using GenAPI for gene presence-absence identification as it performs better on the fragmented genome assemblies than other tools (29).

In summary, we used a genome-wide and hypothesis-free gene presence-absence analysis approach to identify the main patterns of *in vivo* bacterial microevolution. Our analysis adds to the knowledge of how prevalent loss or acquisition of genes is within bacteria evolving in the human host environment and provides a basis to further understand how gene loss and acquisition play a role in host adaptation.

## MATERIALS AND METHODS

### Bacterial isolates, determination of clone types, and lineage definition.

This study used genomic data from a previously reported collection of 474 clinical isolates of P. aeruginosa that were sampled from 34 patients with CF attending the Copenhagen Cystic Fibrosis Center at the University Hospital, Rigshospitalet, Denmark (18). Genomes were sequenced as follows: genomic DNA was prepared from P. aeruginosa isolates on a QIAcube system using a DNeasy blood and tissue kit (Qiagen) and sequenced on an Illumina HiSeq 2000 platform, generating 100-bp paired-end reads and using a multiplexed protocol to obtain an average of 7,139,922 reads (range, 3,111,062 to 13,085,190) for each of the genomic libraries. On average, isolates had estimated genomic coverage of 107× (55× to 195×).

The clone type of each of the isolates was previously reported by Marvig et al. (2015) from a study that determined the clone types by an *ad hoc* analysis (18), and we furthermore confirmed the clone types by the use of Pactyper (https://github.com/MigleSur/Pactyper), which is a tool developed as part of this study for stable and discriminatory clone typing of bacterial genomes. Pactyper was run with default settings, which defined isolates to be of different clone types if they differed by more than 5,000 SNPs in a core genome of 4,760 genes (i.e., all genes shared by 446 of 474 genomes that were successfully assembled *de novo* [see below]). Isolates of the same clone type and from the same patient were defined as being part of the same lineage.

### Bacterial genome assembly.

Sequence reads from each isolate were error corrected and assembled *de novo* by SPAdes version 3.10.1 (51) using k-mer sizes from 21 to 127. Assembled contigs were joined to scaffolds per SPAdes default parameters. *De novo* assemblies of sequence reads from 28 of the 474 isolates (6%) were unsuccessful (>500 scaffolds in the final assembly); thus, those isolates were excluded from the analysis.

### Genome annotation and identification of gene loss and acquisition within lineage pan-genomes.

Genomes assembled *de novo* were annotated using Prokka version 1.11 (52) and a custom annotation database for P. aeruginosa species based on PAO1 (RefSeq assembly accession no. GCF_000006765.1) and UCBPP-PA14 (RefSeq assembly accession no. GCF_000014625.1) reference genomes. GenAPI was run with default settings to determine lineage pan-genomes as well as the presence/absence of genes in individual genomes (29). Note that GenAPI default settings include the specification that genes shorter than 150 nucleotides are excluded from the analysis. Genes identified as absent by GenAPI were confirmed as absent by aligning the raw sequencing reads to the pan-genome with bwa v0.7.15 (53). A total of 52 of 13,246 genes which were identified by GenAPI as absent had ≥50% of the gene covered with ≥10× coverage (mosdepth [54]) and therefore were defined as present in all succeeding analyses.

### Aggregated pan-genome and visualization.

An aggregated pan-genome was determined by gene clustering with GenAPI, which uses CD-HIT-EST version 4.6.1 software (55) and has the requirement for alignments to cover at least 80% of the query gene length and to have a minimum of 90% identity in the alignment. Every gene in the aggregated pan-genome was then aligned back to the individual lineage pan-genomes to determine if the gene was (i) nonpresent in the lineage pan-genome, (ii) present and variable within the lineage, or (iii) present and nonvariable within the lineage. A heat map for the aggregated pan-genome was made with all 45 lineages using R version 3.3.3 (56) and pheatmap library version 1.0.8 (57).

### Identification of the most variable genes.

For a gene to be considered highly variable, it had to be lost or acquired (variable) in at least 4 lineages (among the top 2% of all variable genes). All genes which were identified as highly variable were manually inspected as follows to confirm the results: read sequences from isolates of interest were aligned to the reference PAO1 (RefSeq assembly accession no. GCF_000006765.1) genome using bowtie2 version 2.3.2 (58) with the default parameters for paired-end sequencing. The sequence alignments at genomic positions of interest were visualized with IGV version 2.4.9 (59) and then manually assessed. Genes of non-*Pseudomonas* origin were manually inspected by evaluating their alignments to the pan-genome genes (from the GenAPI analysis).

In total, two genes (PA1352 and PA3457) were concluded to be falsely called as variable because the alignments to the reference PAO1 genome did not support the prediction of the genes being absent. These false calls were in genome regions which are complex and difficult to assemble *de novo*, i.e., calls of gene presence or absence were found to vary with the success of the assembly of the specific genome region rather than as a result of genuine gene presence or absence.

### Reanalysis of P. aeruginosa genomes previously analyzed in another P. aeruginosa study.

Analysis of the data set from a study previously reported by Freschi et al. (2019) (6) included 1,139 of 1,311 genomes as 172 genomes were not publicly available on the day of access (2 March 2019). All available samples were analyzed with SaturnV (https://github.com/ejfresch/saturnV) using the default settings and the “lazy” option and with GenAPI (https://github.com/MigleSur/GenAPI) (29) using the default settings.

### Resistance, virulence, pathoadaptive and prophage origin gene identification.

Resistance, plasmid, and virulence genes were identified by comparing the aggregated pan-genomes of 45 lineages with the corresponding databases by using ABRicate version 0.8 (60). The gene from the corresponding database was considered present if its identity was at least 98% and the alignment made up a minimum of 25% of the gene length.

The PlasmidFinder (61) database (263 sequences; retrieved 21 March 2018) was used for plasmid gene identification, the VFDB database (2,597 genes; retrieved 21 March 2018) (62) was used for virulence gene identification, the Resfinder database (2,280 genes; retrieved 21 March 2018) (63) was used for resistance gene identification, and the ACLAME database (54,945 genes; retrieved 7 June 2018) (64) was used for prophage origin sequence identification. For pathoadaptive gene identification, a list of 52 pathoadaptive genes reported previously by Marvig et al. (2015) (18) was compared to the aggregated pan-genome of the 45 lineages. All isolate assemblies were inspected for the presence of genes in the essential gene list reported previously by Liberati et al. (2006) by using ABRicate version 0.8 (60).

Fisher’s exact test was performed to identify whether the numbers of genes from the corresponding database were significantly different between the within-host variable and nonvariable genes.

### Genomic island identification.

Genomic islands were predicted using the IslandViewer4 (65) webserver with PAO1 (RefSeq assembly accession no. GCF_000006765.1) as the reference genome. Genomic island prediction was performed for the annotated scaffold sequences. IslandViewer4 integrated tools—IslandPath-DIMOB and SIGI-HMM—were used for prediction of genomic islands.

### Pairwise gene and SNP distance estimation between P. aeruginosa isolates.

The gene distance between genomes was defined as the number of genes not present in one of the genomes as determined by GenAPI (i.e., genes present in one genome and absent in the other and vice versa). Pairwise SNP distance was determined using PacTyper (https://github.com/MigleSur/Pactyper), which uses sequence reads to call and compare SNPs across the core genome. The default thresholds of Pactyper require that sequence reads cover at least 80% of the core genome with not less than 10-fold coverage to ensure exclusion of genomes with poor sequencing coverage. The core genome was defined in this study with GenAPI analysis by including all genes shared by the 446 successfully sequenced P. aeruginosa genomes. The core genome contained 4,760 genes (4,705,617 nucleotides).

### Phylogenetic tree generation.

A SNP-based phylogenetic tree was generated with RAxML version 8.2.11 (66) (with the GTRCAT settings for nucleotide sequence analysis and “12345” as a random number seed) by alignments of the previously defined core genome, and PAO1 (RefSeq assembly accession no. GCF_000006765.1) and UCBPP-PA14 (RefSeq assembly accession no. GCF_000014625.1) were included as reference genomes. A SNP-based phylogenetic tree was also generated with Parsnp (67) both with and without the use of a PhiPack (67) recombination filter. A gene presence-absence-based phylogenetic tree was generated with RAxML version 8.2.11 (66) (with the BINCAT settings for nucleotide sequence analysis and “12345” as a random number seed) by using gene presence-absence information from GenAPI analysis, and PAO1 (RefSeq assembly accession no. GCF_000006765.1) and UCBPP-PA14 (RefSeq assembly accession no. GCF_000014625.1) were included as reference genomes. The Microreact webservice was used to visualize the phylogenetic trees (68).

### Data availability.

The sequences analyzed in this work are deposited in the Sequence Read Archive (SRA) under accession no. ERP004853.
