# Supplementary material for: Gene Loss and Acquisition in Lineages of Pseudomonas aeruginosa Evolving in Cystic Fibrosis Patient Airways
Source: mBio. 2020 Oct 27;11(5):e02359-20. doi: 10.1128/mBio.02359-20 (PMC7593970; doi:10.1128/mBio.02359-20)
Supplement: FIG S1 [file mBio.02359-20-sf001.pdf]

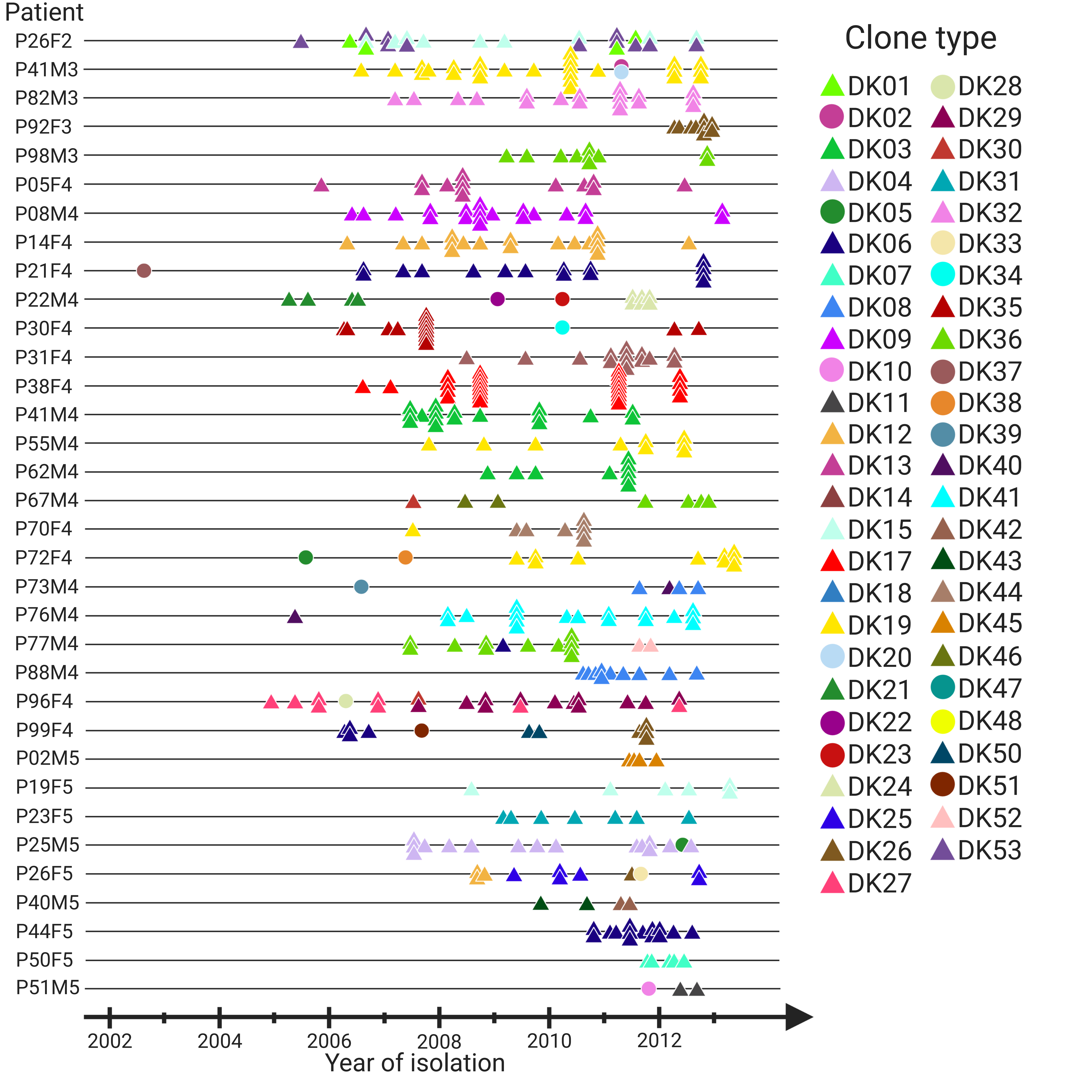

Patient
P26F2
P41M3
P82M3
P92F3
P98M3
P05F4
P08M4
P14F4
P21F4
P22M4
P30F4
P31F4
P38F4
P41M4
P55M4
P62M4
P67M4
P70F4
P72F4
P73M4
P76M4
P77M4
P88M4
P96F4
P99F4
P02M5
P19F5
P23F5
P25M5
P26F5
P40M5
P44F5
P50F5
P51M5
2002
2004
2006
2008
2010
2012
Year of isolation
Clone type
DK01
DK02
DK03
DK04
DK05
DK06
DK07
DK08
DK09
DK10
DK11
DK12
DK13
DK14
DK15
DK17
DK18
DK19
DK20
DK21
DK22
DK23
DK24
DK25
DK26
DK27
DK28
DK29
DK30
DK31
DK32
DK33
DK34
DK35
DK36
DK37
DK38
DK39
DK40
DK41
DK42
DK43
DK44
DK45
DK46
DK47
DK48
DK50
DK51
DK52
DK53
