# Supplementary figures and images for: Gene Loss and Acquisition in Lineages of Pseudomonas aeruginosa Evolving in Cystic Fibrosis Patient Airways
Source: mBio. 2020 Oct 27;11(5):e02359-20. doi: 10.1128/mBio.02359-20 (PMC7593970; doi:10.1128/mBio.02359-20)

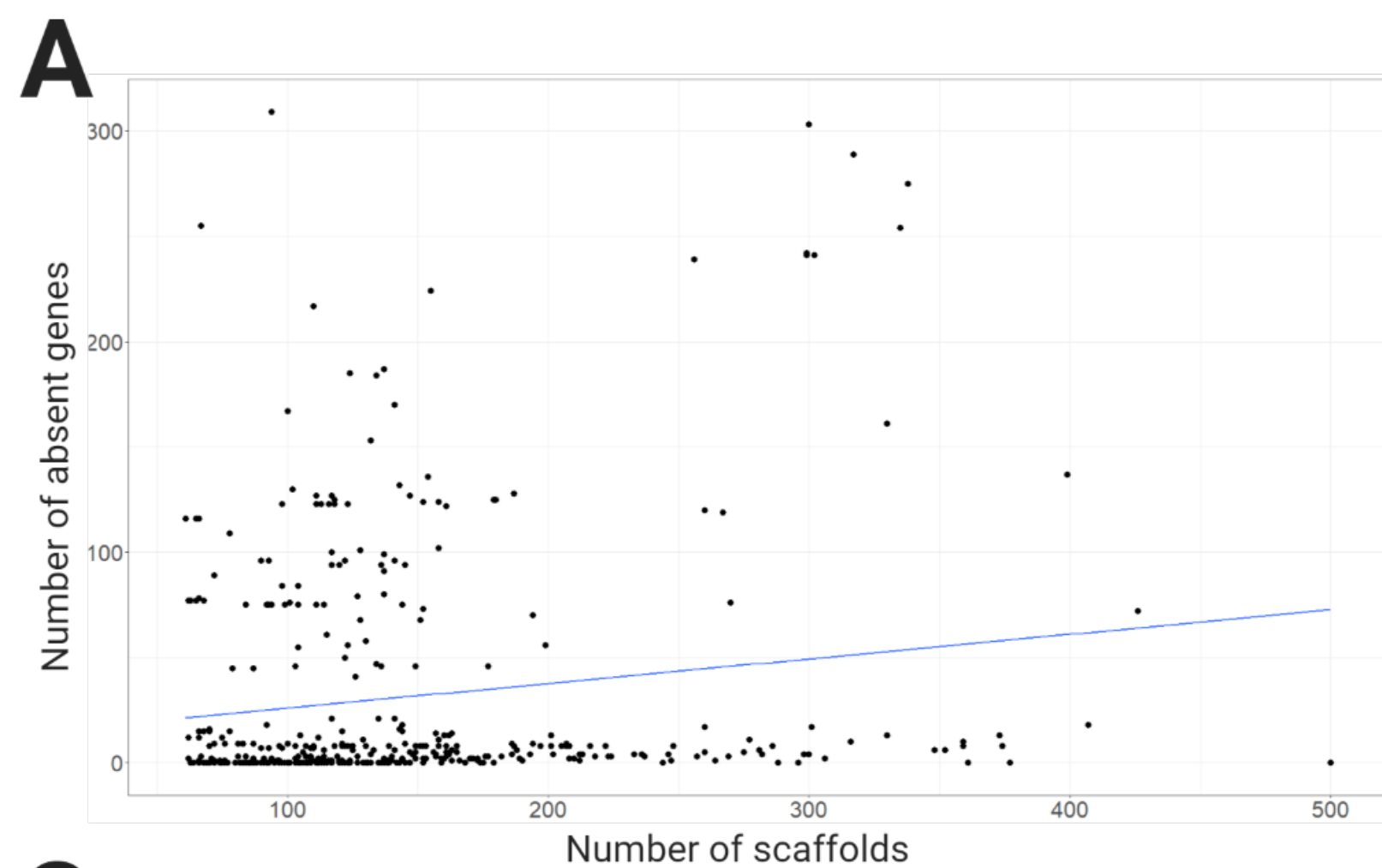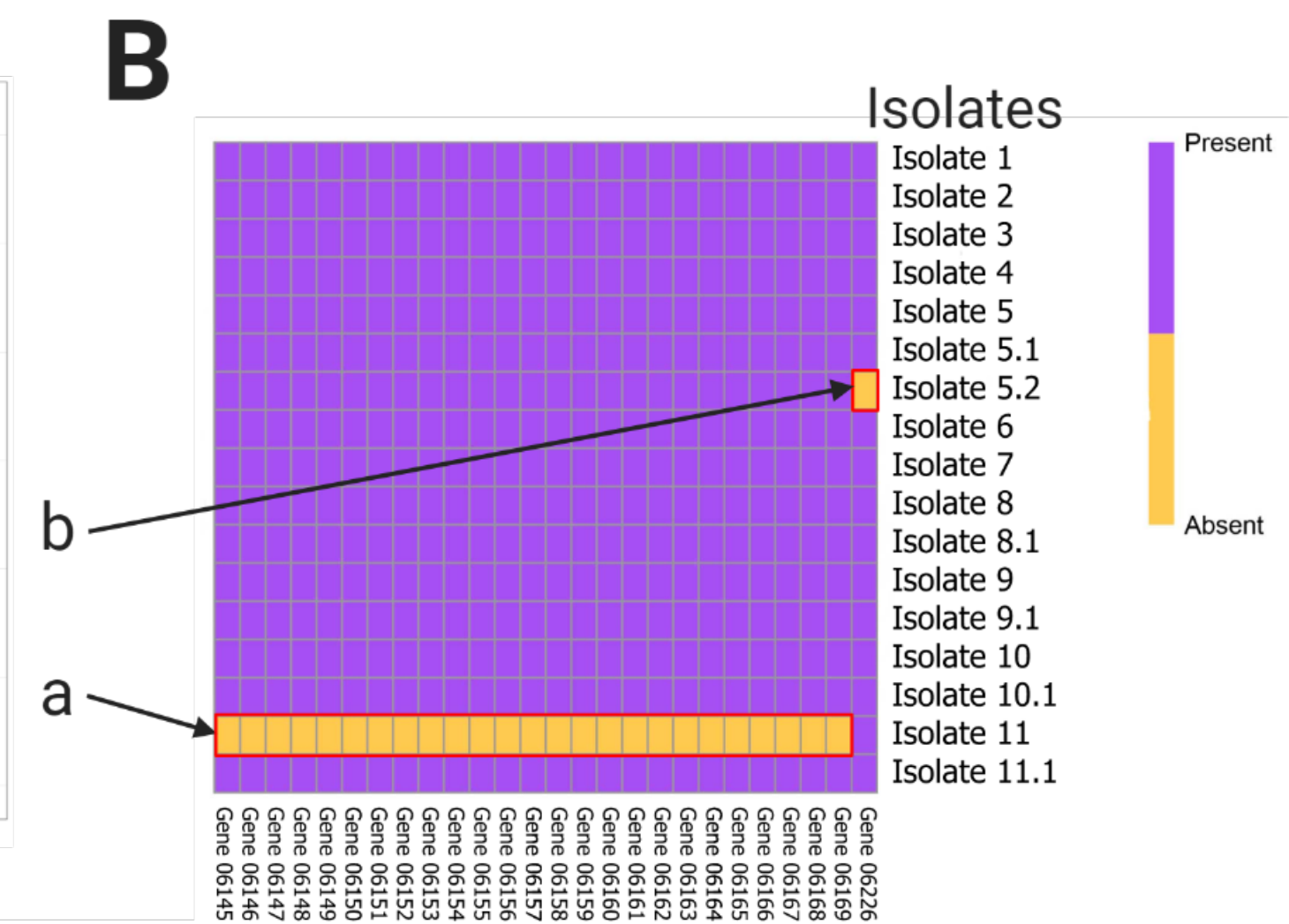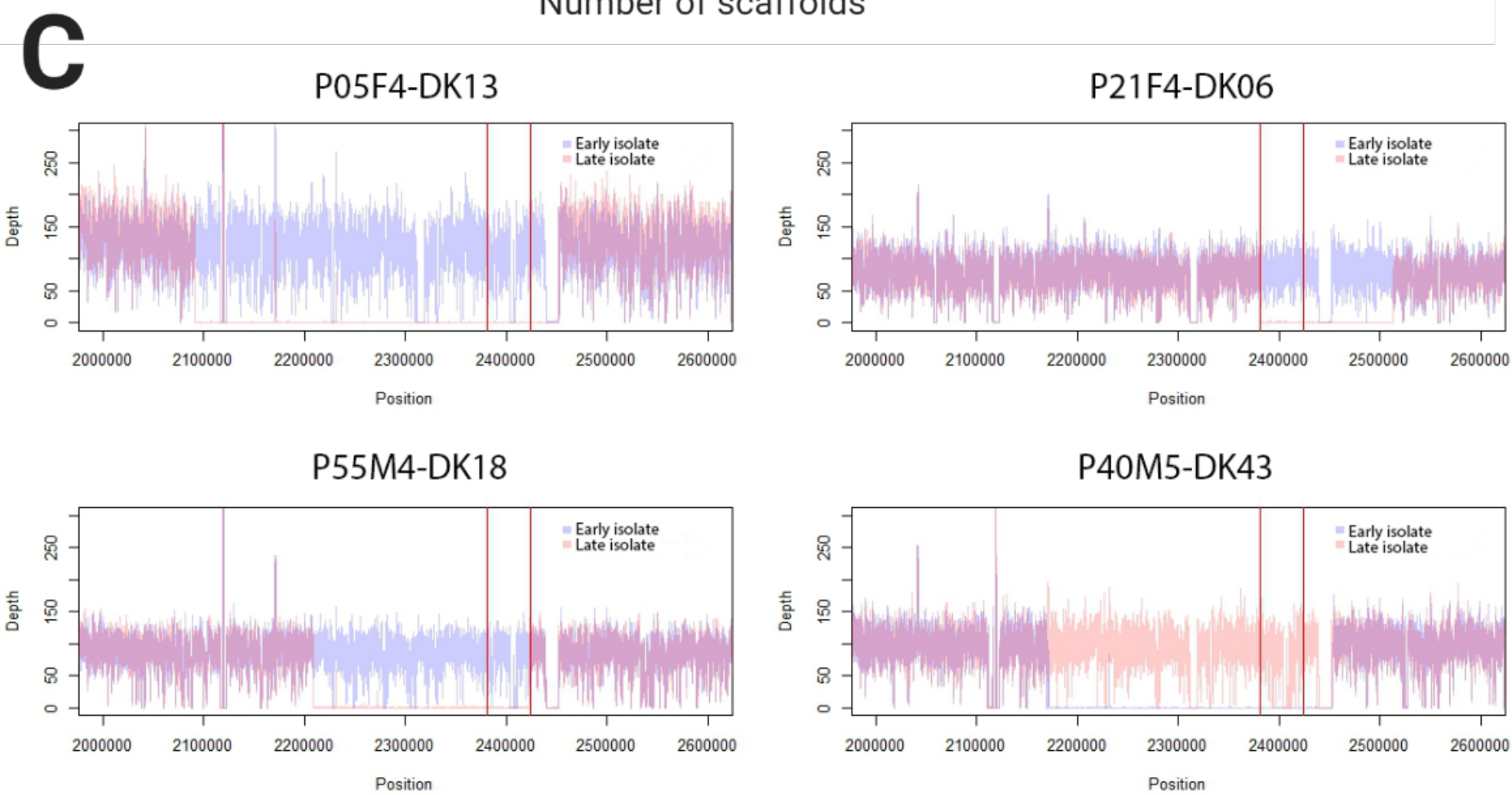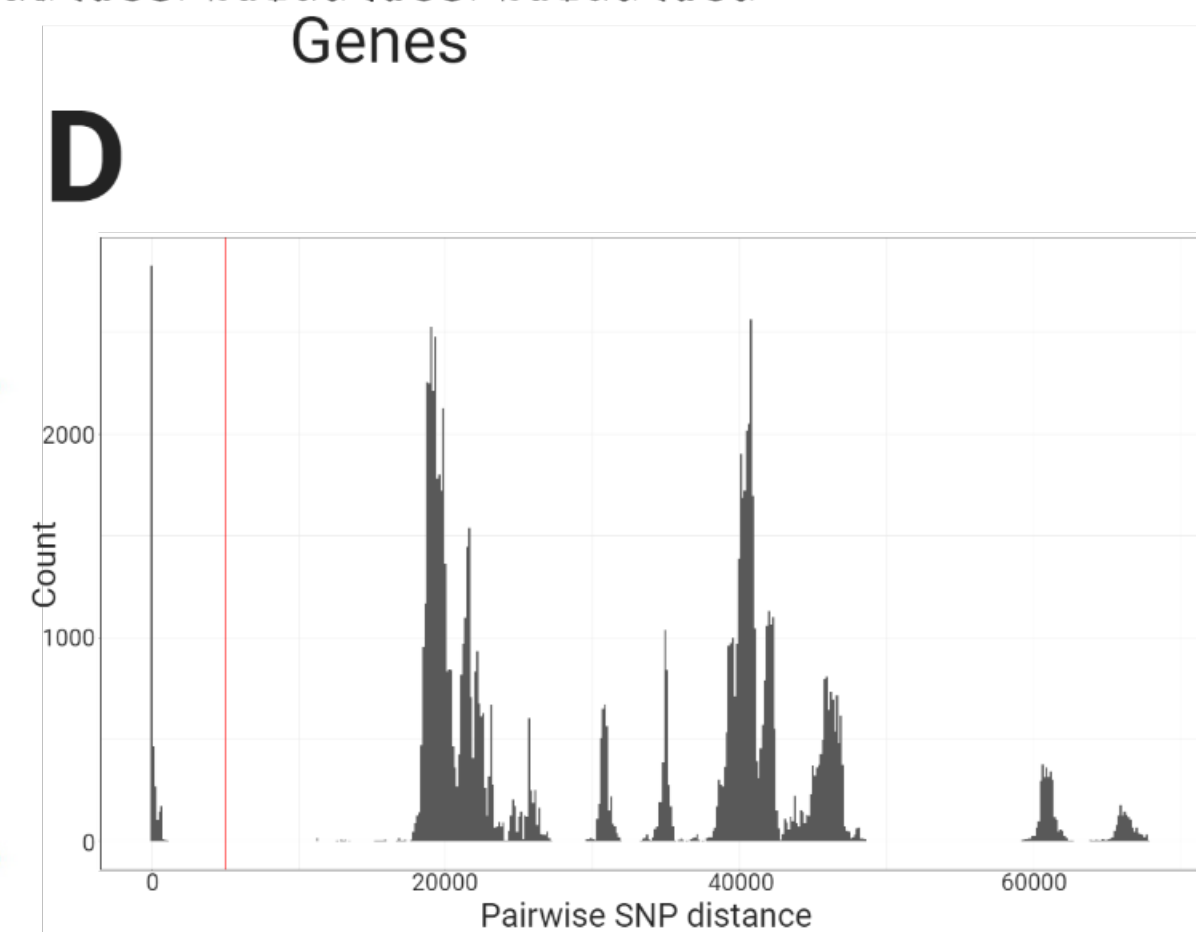

Supplement: FIG S2 [file mBio.02359-20-sf002.pdf]

**A**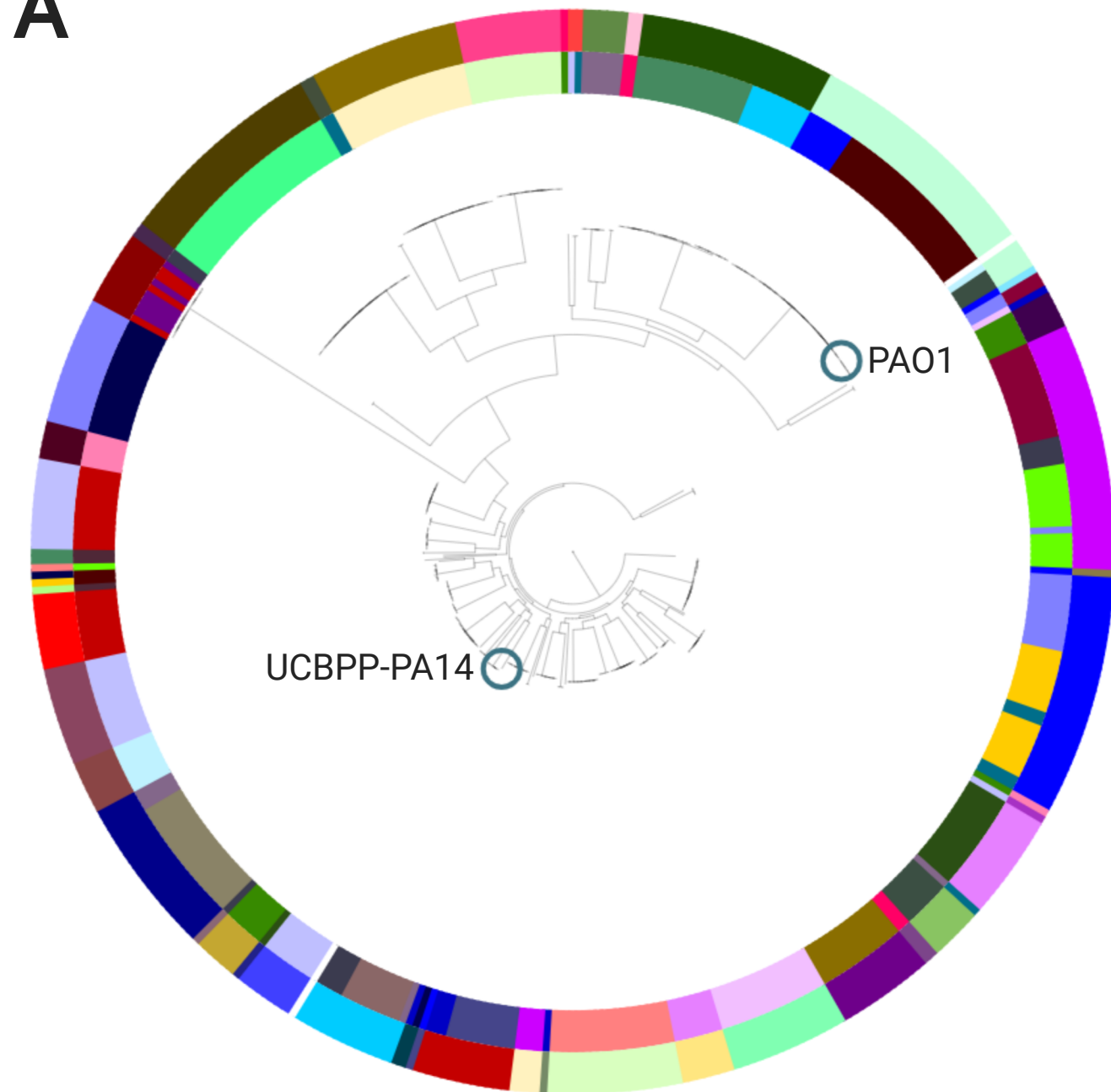**B**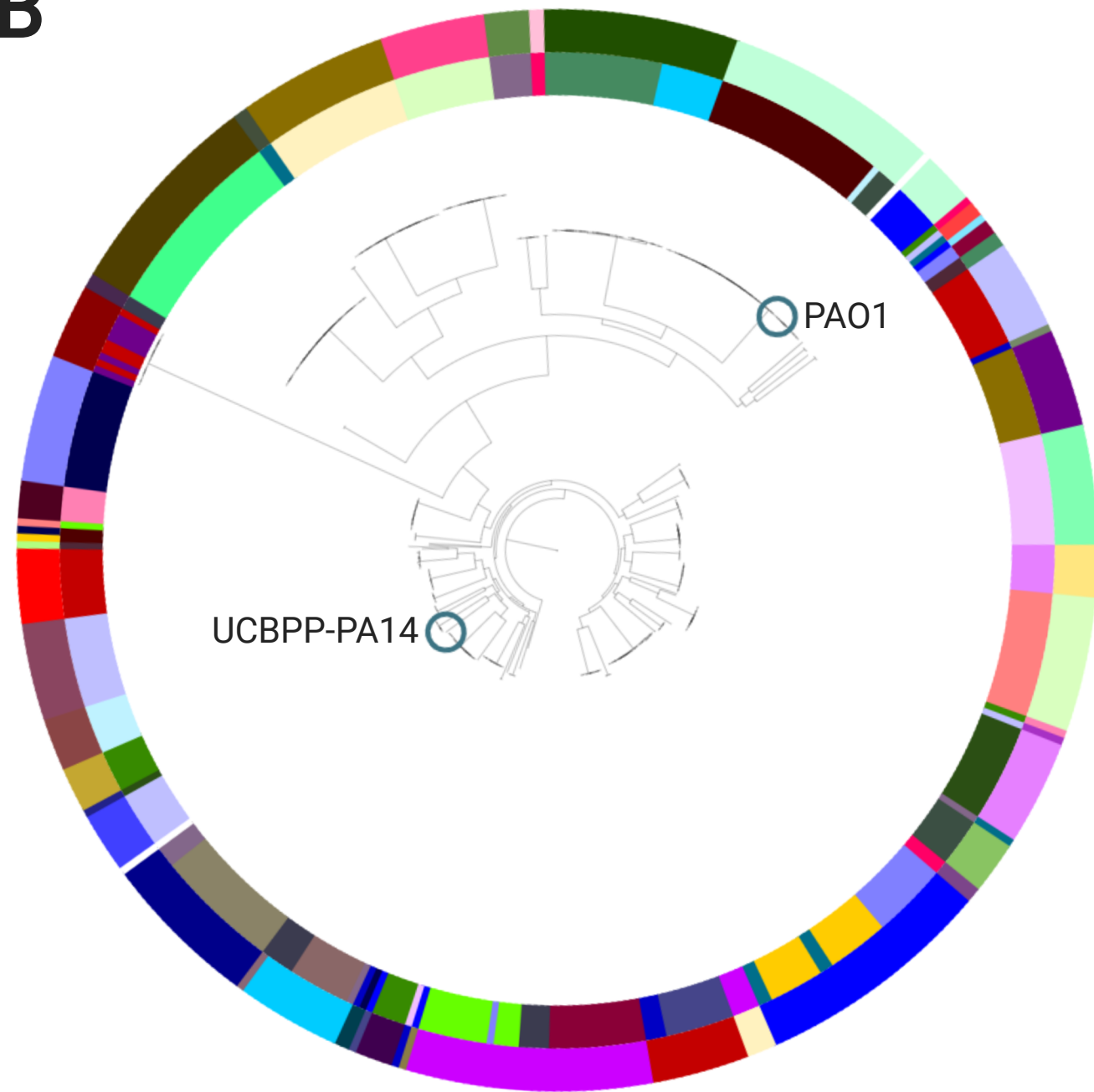

Supplement: FIG S3 [file mBio.02359-20-sf003.pdf]
