# Supplementary material for: Gene Loss and Acquisition in Lineages of Pseudomonas aeruginosa Evolving in Cystic Fibrosis Patient Airways
Source: mBio. 2020 Oct 27;11(5):e02359-20. doi: 10.1128/mBio.02359-20 (PMC7593970; doi:10.1128/mBio.02359-20)
Supplement: TABLE S1 [file mBio.02359-20-st001.docx]

| Lineage | Pan-genome size (without short genes) | Lineage core genome | Variable genes in aggregated core genome | Variable genes in accessory genome | Lineage genes that are part of aggregated accessory genome (unique genes) | Present Resfinder genes (variable) | Present VFDB genes (variable) | Present pathoadaptive genes (variable) |
| --- | --- | --- | --- | --- | --- | --- | --- | --- |
| P36F2-DK01 | 5607 | 5681 | 7 | 19 | 808 (36) | 5 (0) | 227 (0) | 60 (1) |
| P36F2-DK15 | 5722 | 5721 | 0 | 1 | 834 (0) | 5 (0) | 225 (0) | 54 (0) |
| P36F2-DK53 | 5739 | 5727 | 8 | 4 | 839 (39) | 5 (0) | 217 (1) | 55 (0) |
| P41M3-DK19 | 5974 | 5955 | 4 | 15 | 1068 (7) | 5 (0) | 231 (0) | 54 (1) |
| P82M3-DK32 | 5798 | 5797 | 0 | 1 | 909 (42) | 5 (0) | 224 (0) | 54 (0) |
| P92F3-DK26 | 6118 | 6024 | 0 | 94 | 1137 (13) | 5 (0) | 232 (0) | 57 (0) |
| P98M3-DK36 | 6301 | 6092 | 161 | 48 | 1204 (3) | 5 (0) | 229 (2) | 55 (2) |
| P05F4-DK13 | 5675 | 5320 | 232 | 123 | 433 (43) | 5 (0) | 226 (2) | 58 (6) |
| P08M4-DK09 | 5994 | 5727 | 84 | 183 | 839 (140) | 5 (0) | 219 (5) | 50 (1) |
| P14M4-DK12 | 6394 | 6293 | 0 | 101 | 1402 (305) | 5 (0) | 228 (1) | 56 (0) |
| P21F4-DK06 | 6097 | 5854 | 75 | 168 | 967 (4) | 5 (0) | 228 (9) | 56 (0) |
| P22M4-DK21 | 5951 | 5950 | 1 | 0 | 1063 (46) | 5 (0) | 220 (0) | 54 (0) |
| P22M4-DK24 | 5889 | 5844 | 0 | 45 | 957 (119) | 5 (0) | 229 (0) | 55 (0) |
| P30F4-DK35 | 6218 | 6067 | 20 | 131 | 1179 (96) | 5 (0) | 221 (0) | 54 (0) |
| P31F4-DK14 | 5775 | 5773 | 0 | 2 | 886 (78) | 5 (0) | 238 (0) | 51 (1) |
| P38F4-DK17 | 5872 | 5854 | 6 | 12 | 963 (50) | 5 (0) | 225 (1) | 57 (1) |
| P41M4-DK03 | 6079 | 6079 | 0 | 0 | 1191 (167) | 5 (0) | 239 (0) | 53 (0) |
| P55M4-DK18 | 6243 | 5923 | 112 | 208 | 1035 (237) | 5 (0) | 232 (1) | 58 (4) |
| P55M4-DK19 | 6331 | 6314 | 0 | 17 | 1426 (180) | 7 (0) | 235 (0) | 51 (0) |
| P62M4-DK03 | 6140 | 6054 | 0 | 86 | 1167 (234) | 5 (0) | 229 (0) | 54 (1) |
| P67M4-DK36 | 6291 | 6276 | 0 | 15 | 1387 (0) | 5 (0) | 229 (0) | 56 (0) |
| P67M4-DK46 | 6170 | 6040 | 0 | 130 | 1153 (230) | 6 (0) | 220 (0) | 52 (0) |
| P70F4-DK44 | 5983 | 5886 | 8 | 89 | 999 (222) | 5 (0) | 222 (0) | 56 (1) |
| P72F4-DK19 | 6089 | 6014 | 1 | 74 | 1127 (115) | 5 (0) | 228 (0) | 53 (0) |
| P73M4-DK08 | 5636 | 5636 | 0 | 0 | 748 (19) | 5 (0) | 219 (0) | 54 (0) |
| P76M4-DK41 | 5747 | 5628 | 0 | 119 | 741 (59) | 5 (0) | 224 (0) | 56 (0) |
| P77F4-DK36 | 7008 | 6535 | 24 | 449 | 1648 (540) | 5 (0) | 233 (5) | 55 (2) |
| P77F4-DK52 | 5839 | 5839 | 0 | 0 | 952 (63) | 5 (0) | 238 (0) | 54 (0) |
| P88M4-DK08 | 5807 | 5760 | 43 | 4 | 873 (95) | 5 (0) | 218 (0) | 53 (3) |
| P96F4-DK27 | 6272 | 6105 | 0 | 167 | 1218 (174) | 5 (0) | 233 (0) | 54 (0) |
| P96F4-DK29 | 5649 | 5634 | 0 | 15 | 747 (78) | 5 (0) | 227 (0) | 56 (0) |
| P99F4-DK06 | 6163 | 6163 | 0 | 0 | 1276 (80) | 5 (0) | 224 (0) | 53 (0) |
| P99F4-DK26 | 6526 | 6431 | 0 | 95 | 1543 (61) | 5 (0) | 237 (0) | 59 (0) |
| P99F4-DK50 | 5707 | 5705 | 0 | 2 | 818 (48) | 5 (0) | 230 (0) | 55 (0) |
| P02M5-DK45 | 5964 | 5740 | 0 | 224 | 853 (285) | 5 (0) | 223 (0) | 57 (0) |
| P19F5-DK15 | 5724 | 5680 | 1 | 43 | 793 (4) | 5 (0) | 225 (0) | 55 (1) |
| P23F5-DK31 | 5940 | 5940 | 0 | 0 | 1052 (83) | 5 (0) | 231 (0) | 57 (0) |
| P25M5-DK04 | 6016 | 5859 | 1 | 156 | 972 (143) | 5 (0) | 231 (0) | 54 (0) |
| P26F5-DK12 | 5723 | 5723 | 0 | 0 | 836 (59) | 5 (0) | 229 (0) | 55 (0) |
| P26F5-DK25 | 6027 | 5970 | 0 | 57 | 1082 (160) | 5 (0) | 231 (0) | 53 (1) |
| P40M5-DK42 | 6407 | 6407 | 0 | 0 | 1520 (206) | 5 (0) | 222 (0) | 56 (0) |
| P40M5-DK43 | 6085 | 5830 | 144 | 111 | 943 (161) | 5 (0) | 231 (1) | 56 (4) |
| P44F5-DK06 | 6089 | 6076 | 2 | 11 | 1186 (100) | 5 (0) | 225 (2) | 54 (1) |
| P50F5-DK07 | 5713 | 5711 | 0 | 2 | 824 (57) | 5 (0) | 224 (0) | 53 (0) |
| P51M5-DK11 | 5808 | 5808 | 0 | 0 | 921 (51) | 5 (0) | 214 (0) | 53 (0) |
