# Supplementary material for: Gene Loss and Acquisition in Lineages of Pseudomonas aeruginosa Evolving in Cystic Fibrosis Patient Airways
Source: mBio. 2020 Oct 27;11(5):e02359-20. doi: 10.1128/mBio.02359-20 (PMC7593970; doi:10.1128/mBio.02359-20)
Supplement: TABLE S2 [file mBio.02359-20-st002.docx]

| **Gene name** | **PseudoCAP function** |
| --- | --- |
| PA0637 | Related to phage, transposon, or plasmid |
| PA2565 | Hypothetical, unclassified, unknown |
| PA2387 | Transcriptional regulators; Transport of small molecules |
| PA1931 | Energy metabolism; Carbon compound catabolism |
| PA2030 | Hypothetical, unclassified, unknown |
| PA0050 | Hypothetical, unclassified, unknown |
| PA0818 | Hypothetical, unclassified, unknown |
| PA0971 | Membrane proteins, Transport of small molecules |
| PA2288 | Hypothetical, unclassified, unknown |
| PA2341 | Transport of small molecules |
| PA2048 | Hypothetical, unclassified, unknown |
| PA2143 | Hypothetical, unclassified, unknown |
| PA2149 | Hypothetical, unclassified, unknown |
| PA2056 | Transcriptional regulators |
| PA2166 | Hypothetical, unclassified, unknown |
| PA2169 | Hypothetical, unclassified, unknown |
| PA2271 | Putative enzymes |
| PA2273 | Transcriptional regulators |
| PA2279 | Transport of small molecules; Adaptation, Protection |
| PA1966 | Putative enzymes |
| PA1981 | Hypothetical, unclassified, unknown |
| PA2094 | Transcriptional regulators; Membrane proteins |
| PA3441 | Transport of small molecules |
| PA1924 | Hypothetical, unclassified, unknown |
| PA1394 | Hypothetical, unclassified, unknown |
| PA1879 | Hypothetical, unclassified, unknown |
| PA1884 | Transcriptional regulators |
| PA2468 | Transcriptional regulators |
| PA2491 | Transcriptional regulators; Putative enzymes |
