# Supplementary material for: Gene Loss and Acquisition in Lineages of Pseudomonas aeruginosa Evolving in Cystic Fibrosis Patient Airways
Source: mBio. 2020 Oct 27;11(5):e02359-20. doi: 10.1128/mBio.02359-20 (PMC7593970; doi:10.1128/mBio.02359-20)
Supplement: TABLE S3 [file mBio.02359-20-st003.docx]

|  | Number of total variable genes | Number of lost genes | Number of genes variable in a group | Number of genes variable individually | Number of variable prophage genes |
| --- | --- | --- | --- | --- | --- |
| P36F2-DK01 | 26 | 23 | 23 | 3 | 4 |
| P36F2-DK15 | 1 | 1 | 0 | 1 | 0 |
| P36F2-DK53 | 12 | 10 | 5 | 7 | 0 |
| P41M3-DK19 | 19 | 18 | 14 | 5 | 0 |
| P82M3-DK32 | 1 | 1 | 0 | 1 | 0 |
| P92F3-DK26 | 94 | 94 | 94 | 0 | 9 |
| P98M3-DK36 | 209 | 209 | 205 | 4 | 4 |
| P05F4-DK13 | 355 | 355 | 349 | 6 | 0 |
| P08M4-DK09 | 267 | 220 | 260 | 7 | 67 |
| P14M4-DK12 | 101 | 95 | 68 | 33 | 7 |
| P21F4-DK06 | 243 | 238.5 | 242 | 1 | 28 |
| P22M4-DK21 | 1 | 0 | 0 | 1 | 0 |
| P22M4-DK24 | 45 | 45 | 45 | 0 | 1 |
| P30F4-DK35 | 151 | 145 | 148 | 3 | 40 |
| P31F4-DK14 | 2 | 2 | 0 | 2 | 0 |
| P38F4-DK17 | 18 | 6 | 7 | 11 | 0 |
| P41M4-DK03 | 0 | 0 | 0 | 0 | 0 |
| P55M4-DK18 | 320 | 320 | 310 | 10 | 68 |
| P55M4-DK19 | 17 | 17 | 17 | 0 | 2 |
| P62M4-DK03 | 86 | 10 | 82 | 4 | 3 |
| P67M4-DK36 | 15 | 3 | 14 | 1 | 2 |
| P67M4-DK46 | 130 | 130 | 130 | 0 | 45 |
| P70F4-DK44 | 97 | 97 | 97 | 0 | 0 |
| P72F4-DK19 | 75 | 75 | 71 | 4 | 17 |
| P73M4-DK08 | 0 | 0 | 0 | 0 | 0 |
| P76M4-DK41 | 119 | 105.5 | 118 | 1 | 55 |
| P77F4-DK36 | 473 | 456 | 448 | 25 | 0 |
| P77F4-DK52 | 0 | 0 | 0 | 0 | 0 |
| P88M4-DK08 | 47 | 47 | 45 | 2 | 0 |
| P96F4-DK27 | 167 | 167 | 165 | 2 | 21 |
| P96F4-DK29 | 15 | 10 | 14 | 1 | 2 |
| P99F4-DK06 | 0 | 0 | 0 | 0 | 0 |
| P99F4-DK26 | 95 | 88 | 95 | 0 | 32 |
| P99F4-DK50 | 2 | 0 | 2 | 0 | 0 |
| P02M5-DK45 | 224 | 223 | 220 | 4 | 0 |
| P19F5-DK15 | 44 | 41 | 41 | 3 | 2 |
| P23F5-DK31 | 0 | 0 | 0 | 0 | 0 |
| P25M5-DK04 | 157 | 149 | 153 | 4 | 3 |
| P26F5-DK12 | 0 | 0 | 0 | 0 | 0 |
| P26F5-DK25 | 57 | 2 | 55 | 2 | 49 |
| P40M5-DK42 | 0 | 0 | 0 | 0 | 0 |
| P40M5-DK43 | 255 | 0 | 254 | 1 | 0 |
| P44F5-DK06 | 13 | 8 | 5 | 5 | 1 |
| P50F5-DK07 | 2 | 0 | 2 | 0 | 0 |
| P51M5-DK11 | 0 | 0 | 0 | 0 | 0 |
