# Supplementary material for: Gene Loss and Acquisition in Lineages of Pseudomonas aeruginosa Evolving in Cystic Fibrosis Patient Airways
Source: mBio. 2020 Oct 27;11(5):e02359-20. doi: 10.1128/mBio.02359-20 (PMC7593970; doi:10.1128/mBio.02359-20)
Supplement: TABLE S4 [file mBio.02359-20-st004.docx]

|  | Number of genes variable | Cumulative percent of all variable genes (%) |
| --- | --- | --- |
| Genes variable in 0 lineages | 11,644 | **-** |
| Genes variable in 1 lineage | 2,117 | 100.0 |
| Genes variable in 2 lineages | 362 | 24.9 |
| Genes variable in 3 lineages | 284 | 12.0 |
| Genes variable in 4 lineages | 48 | 2.0 |
| Genes variable in 5+ lineages | 7 | 0.2 |
