# Supplementary material for: Gene Loss and Acquisition in Lineages of Pseudomonas aeruginosa Evolving in Cystic Fibrosis Patient Airways
Source: mBio. 2020 Oct 27;11(5):e02359-20. doi: 10.1128/mBio.02359-20 (PMC7593970; doi:10.1128/mBio.02359-20)
Supplement: TABLE S5 [file mBio.02359-20-st005.docx]

| Lineage | Gene name | Reference (DOI) |
| --- | --- | --- |
| P08M4-DK09 | AlgR | 10.1128/IAI.70.11.6083-6093.2002 |
| P08M4-DK09 | Clp protease proteolytic subunit | 10.1016/j.resmic.2009.08.017 |
| P30F4-DK35 | LpxO | 10.1177/1753425909106436 |
| P30F4-DK35 | PrtR | 10.1128/IAI.01388-13 |
| P67M4-DK46 | Clp protease proteolytic subunit | 10.1016/j.resmic.2009.08.017 |
| P67M4-DK46 | PrtR | 10.1128/IAI.01388-13 |
| P72F4-DK19 | PrtR | 10.1128/IAI.01388-13 |
| P72F4-DK19 | AlgR | 10.1128/IAI.70.11.6083-6093.2002 |
| P72F4-DK19 | ClpX | 10.1099/mic.0.2008/017368-0 |
| P72F4-DK19 | AlgI | 10.1128/CMR.4.2.191 |
| P76M4-DK41 | Clp protease proteolytic subunit | 10.1016/j.resmic.2009.08.017 |
| P21F4-DK06 | FimU | 10.1073/pnas.1415712111 |
| P21F4-DK06 | Clp protease proteolytic subunit | 10.1016/j.resmic.2009.08.017 |
| P30F4-DK35 | PrtR | 10.1128/IAI.01388-13 |
| P30F4-DK35 | Clp protease proteolytic subunit | 10.1016/j.resmic.2009.08.017 |
| P55M4-DK18 | PrtR | 10.1128/IAI.01388-13 |
| P55M4-DK18 | Clp protease proteolytic subunit | 10.1016/j.resmic.2009.08.017 |
| P96F4-DK27 | PrtR | 10.1128/IAI.01388-13 |
| P96F4-DK27 | Clp protease proteolytic subunit | 10.1016/j.resmic.2009.08.017 |
