# Supplementary material for: Gene Loss and Acquisition in Lineages of Pseudomonas aeruginosa Evolving in Cystic Fibrosis Patient Airways
Source: mBio. 2020 Oct 27;11(5):e02359-20. doi: 10.1128/mBio.02359-20 (PMC7593970; doi:10.1128/mBio.02359-20)
Supplement: TEXT S1 [file mBio.02359-20-s0001.docx]

Isolate clone types were previously defined by Marvig *et al.* (2015) [1] by one-to-one pairwise alignment of genomes, and genomes that differed by >10,000 SNPs were defined as of different clone types. This way of defining clone types was ambiguous because the number of SNPs was determined on gene alignments shared between only the two respective genomes rather than based on a fixed set of genes. Therefore, we employed the identified core genome in a more robust method to define clone types, and using the core genome as a fixed basis for determining SNP distances, we found that 5,000 SNPs clearly confirmed and defined clone types (Figure S2D). Furthermore, we developed the method into a workflow—Pactyper (<https://github.com/MigleSur/Pactyper>) [2]—that can be used to define clone types from the core genome and raw sequencing data of any collection of bacterial isolates. Pactyper takes a pre-defined core genome and sample sequencing reads (FASTQ) as an input. The tool continuously, with every new submitted genome, builds a matrix of pairwise core-genome SNP distances between isolates and assigns a clone type to the submitted isolate. If the SNP distance is lower than the clone type threshold (default: 5000 SNPs), the existing clone type which passes the criteria is assigned. If none of the clone types pass the criteria when compared with the input sample, a new clone type is assigned.

Such typing approach has its set of advantages and disadvantages. Pactyper is based on core genome SNPs (cgSNP) as opposed to multilocus sequence typing (MLST) which is based on housekeeping genes; thus, the discriminatory power of Pactyper is considerably increased. Emerging core-genome MLST (cgMLST) and whole-genome MLST (wgMLST) typing approaches are highly discriminatory and comparable to the cgSNP-based approaches [3, 4]. Furthermore, cgSNP-based methods prove higher discriminatory power than cgMLST-based approaches in bacteria where little recombination has occurred [5]. Another advantage of Pactyper is by including new samples, the local database increases and becomes more accurate. Continuously updated distance matrix serves as the main advantage of Pactyper for application in a clinical set up when locally following outbreaks or lineages of chronic bacterial infections. Contrarily, cgMLST is more easily scalable and often equally accurate with the advantage of highly standardized typing schemes for conventional microorganisms, allowing direct comparison between datasets [3, 6]. Ultimately, we developed a tool—Pactyper—that meets the need for a standardized way to define clone types and quantify genome SNP distances at the population level using whole genome sequencing (WGS) reads.

# **References**

| [1] | R. L. Marvig, L. M. Sommer, S. Molin and H. K. Johansen, "Convergent evolution and adaptation of Pseudomonas aeruginosa within patients with cystic fibrosis," *Nature Genetics,* 2015. |
| --- | --- |
| [2] | M. Gabrielaite and R. L. Marvig, "Pactyper: Snakemake pipeline for continuous clone type prediction for WGS sequenced bacterial isolates based on their core genome," 2020. [Online]. Available: https://github.com/MigleSur/Pactyper. |
| [3] | R. O. de Sales, L. B. Migliorini, R. Puga, B. Kocsis and P. Severino, "A Core Genome Multilocus Sequence Typing Scheme for Pseudomonas aeruginosa," *Frontiers in Microbiology,* vol. 11, 26 5 2020. |
| [4] | M. E. Pearce, N. F. Alikhan, T. J. Dallman, Z. Zhou, K. Grant and M. C. Maiden, "Comparative analysis of core genome MLST and SNP typing within a European Salmonella serovar Enteritidis outbreak," *International Journal of Food Microbiology,* vol. 274, pp. 1-11, 2 6 2018. |
| [5] | E. Miro, J. W. Rossen, M. A. Chlebowicz, D. Harmsen, S. Brisse, V. Passet, F. Navarro, A. W. Friedrich and S. García-Cobos, "Core/Whole Genome Multilocus Sequence Typing and Core Genome SNP-Based Typing of OXA-48-Producing Klebsiella pneumoniae Clinical Isolates From Spain," *Frontiers in Microbiology,* vol. 10, 31 1 2020. |
| [6] | "cgMLST.org Nomenclature Server," [Online]. Available: https://www.cgmlst.org/ncs. |
