# Supplementary material for: Gene Loss and Acquisition in Lineages of Pseudomonas aeruginosa Evolving in Cystic Fibrosis Patient Airways
Source: mBio. 2020 Oct 27;11(5):e02359-20. doi: 10.1128/mBio.02359-20 (PMC7593970; doi:10.1128/mBio.02359-20)
Supplement: TABLE S6 [file mBio.02359-20-st006.docx]

| Lineage | Loss of Clp protease or *ptrR* genes | Loss of *hcnABC* loci and *exoY* gene |
| --- | --- | --- |
| P05F4-DK13 | No | Yes |
| P40M5-DK43 | No | Yes |
| P08M4-DK09 | Yes | No |
| P30F4-DK35 | Yes | No |
| P67M4-DK46 | Yes | No |
| P72F4-DK19 | Yes | No |
| P76M4-DK41 | Yes | No |
| P96F4-DK27 | Yes | No |
| P21F4-DK06 | Yes | Yes |
| P55M4-DK18 | Yes | Yes |
